# Supplementary material for: Effects of speculum lubrication on cervical smears for cervical cancer screening: A double blind randomized clinical trial
Source: PLoS One. 2024 May 24;19(5):e0292207. doi: 10.1371/journal.pone.0292207 (PMC11125561; doi:10.1371/journal.pone.0292207)
Supplement: S2 File — (DOCX) [file pone.0292207.s003.docx]

**EFFECTS OF SPECULUM LUBRICATION ON CERVICAL SMEARS FOR CERVICAL CANCER SCREENING: A DOUBLE-BLIND RANDOMIZED CONTROLLED TRIAL**

**A STUDY PROTOCOL**

**TABLE OF CONTENTS**

TITLE PAGE……………………………….………………………….…………………...…1

TABLE OF CONTENTS ………………………….…………………….………………..….2

ABSTRACT ……………………………………………………..…….………………….…5

CHAPTER ONE: INTRODUCTION……………………………….….……………..………7

CHAPTER TWO: LITERATURE REVIEW………………….……..………………………..11

2.1 Search Strategy ………………..…………………………….….………………………...12

2.2 Screening Options for Cancer of the Cervix …………………….……………….……….12

2.3 Options of Speculum Lubrication ……………………….……….………...….…….……13

2.4 Speculum lubrication and adequacy of cervical smears ……..….…………….……………15

2.5 Speculum lubrication for cervical smears and pain perception……….……………………18

2.6 Speculum lubrication and willingness for repeat cervical cancer screening…………..……20

2.7 Conclusion of Literature Review……………………………………………………………21

CHAPTER THREE: AIM AND OBJECTIVES ………………………………….……………24

CHAPTER FOUR: RESEARCH METHODOLOGY ………………………….……………..26

4.1 Study Design…………………………………………………………….…….…………..26

4.2 Study Setting…………………………………………………………….……….………..26

4.3 Sample Size Determination………………………………………….……….…………...27

4.4 Ethical Considerations ………………………………………………………….…….....28

4.5 Study Population………………………………………………………………….……....28

4.6 Inclusion Criteria………………………………………………………………....……….29

4.7 Exclusion Criteria………………………………………………………..………….…….29

4.8 Data Collection……………………………………………………….…….………....…..29

4.9 Sampling Approach, Randomization and Allocation Sequence……….……..…….….….29

4.10 Study Procedures ………………………………………………….………..……....…..30

4.11 Outcome Measures...………………………………………………….…….….……..…..32

4.12 Planned Handling of Results…………………………………………….…….…….……32

4.12.1 Data Analysis………………….…………………………………….……….…....……32

4.13 Strengths and Limitations of the Study ……………………………….……….……....…36

4.14 Conflict of Interest …………………………………………………….……..…………..36

APPENDIX 1: CONSENT FORM ……………………………………….………..………….38

APPENDIX 2: RESEARCH PROFORMA………………………...………………..….…….41

REFERENCES…………………………………………………………………………..…….44

**ABSTRACT**

**Background:** Carcinoma of the cervix is the most prevalent gynaecological malignancy in our environment, and it is associated with significant morbidity and mortality. Papanicolaou test is a well-recognised and efficient screening modality but the uptake of Pap smear in our environment remains poor. Fear of pain from speculum insertion during vaginal examination has been noted as a factor that may reduce the compliance of women to cervical cancer screening and repeat testing. Speculum lubrication may help to reduce these pain and discomfort, but there are fears of its interference with cytological results.

**Aim and Objectives:** To determine and compare adequacy of cervical cytology smears and mean pain scores of women undergoing cervical screening with or without speculum lubrication.

**Research Methodology:** This proposed work will be a randomized controlled study among women having cervical cancer screening at the gynaecological clinic of Nnamdi Azikiwe University Teaching Hospital, Nnewi. Sixty-six patients would be randomly assigned to the ‘Gel group’ and another 66 patients to the ‘No Gel’ group. Samples will be collected from each participant for Pap smear with lubricated vaginal speculum for the ‘Gel group’ and non-lubricated vaginal speculum for the ‘No Gel group’. Pain scores will be assessed with the numeric rating scale for pain. The primary outcome measures will be the mean numeric rating scale pain scores and proportion of women with inadequate cervical cytology smears.

**Handling of Results:** The Statistical Packages for Social Sciences (SPSS) would be employed for data analysis. Statistical test of significance would be deduced at p-value less than 0.05.

**Strengths and Limitations: T**his proposed study is the first of its kind in Nigeria. Conclusions may not be generalized to other types of lubricant gels since only one type of lubricant gel will be used. The histopathologist and the study participants will be blinded hence double-blind.

**CHAPTER ONE**

**INTRODUCTION**

Cancer of the cervix ranks fourth in the list of the most prevalent malignancies in females with a projected 570 000 incidence in 2018. ^1^ It represents 6.6 per cent of all gynaecological carcinomas and is responsible for 7.5 per cent of all female malignancy mortality worlwide.^1^ Out of the 311 000 cases of mortality estimated from carcinoma of the cervix yearly, greater than 85% of these deaths happen in areas that are less developed.^1^

Cervical carcinoma is the predominant gynaecological malignancy occurring in females of sub-Saharan African region ^4^ with a projected 70,722 incidence annually,^5^ while it comes second in Nigeria with breast cancer topping the list of malignancies in women. ^6,7,8^ Previous studies have shown that 53.1 million females older than 15 years have increased chances of having carcinoma of the cervix in Nigeria.^9^ There are 14,943 people diagnosed yearly in Nigeria with 10,403 deaths and an incidence of 15.5 per 100,000 women. ^9^

Introducing screening for cancer of the cervix and improving its uptake in developing countries is a key factor in decreasing burdens due to deaths from malignancy of the cervix. The usage of screening programs can be improved via increasing sensitization on factors that increase chances of having carcinoma of the cervix which include many sex partners, early coitarche, multiparity, HPV infections, early pregnancy, extensive use of oral contraceptives and HIV infections.^10^

Screening methods for cancer of the cervix include the conventional Papanicolaou test, colposcopy, Visual Inspection of the cervix with Lugol’s iodine and Acetic acid and Human papilloma virus testing .^11^ Presently there are 3 kinds of vaccines available against HPV infection namely Cervarix, Gardasil 4, and Gardasil 9. ^12,13^

In high income countries, there are well structured programs which enable vaccination of girls against HPV and regular screening of women resulting in remarkable decrease in the development and complications of carcinoma of the cervix.^14,15,16^ Screening helps to achieve early identification and intervention which prevents up to 80% of cervical malignancies in high income countries,^14,15,16^ whereas in poor income countries, there is poor access to vaccination and testing resulting in diagnosis of cervical cancer mainly in advanced stages.^2,3^ In addition, these countries lack access to facilities required for the management of such advanced stage diseases giving rise to a greater death rate from malignancy of the cervix in poor countries.^2,3^

In as much as efforts are been made to improve vaccinations for HPV in order to prevent carcinoma of the cervix, early identification of premalignant lesions of the cervix through cervical Papanicolaou smear cytology screening remains a key factor for achieving a decline in the development and complications of carcinoma of the cervix in poor income countries where vaccination for Human Papilloma Virus services is limited.^18^

The Papanicolaou test is a well-recognized, efficient and reliable tool employed in early identification of premalignant lesions of the cervix resulting in substantial decrease in disease burden of carcinoma of the cervix.^19^ It is cost-effective and the technique is simple. Despite this, the uptake of Papanicolaou smear in our environment remains poor.^2^ Pain and discomfort associated with examination of the vagina can discourage women from assessing regular test.^20^ Other factors that may hinder compliance include lack of awareness, cost implication, anxiety and cultural beliefs.^40^

Insertion of speculum for examination of vagina is an important factor responsible for non- compliance to regular screening and repeat testing for carcinoma of the cervix because of the embarrassment, anxiety, pain and discomfort associated with it.^21^  In Australia, a research seeking to find out the attitude of women concerning self-insertion compared to physician insertion of the speculum showed that 91% of the study population will prefer to insert the speculum by themselves rather than a physician doing it because of the embarrassment and discomfort associated with it. ^22^

During intercourse, lubrication is physiologically essential for easy penetration of the vagina and absence of optimal lubrication results in dyspareunia. So we cannot justify inserting without lubrication a rigid instrument like a speculum into the vagina. It is the duty of gynaecologists to alleviate the pain and discomfort encountered in vaginal examinations with speculum to prevent the unfavourable effects of women refusing vaginal examinations due to pain. Speculum lubrication should be employed to minimize discomfort and pain during vaginal examinations thereby ultimately increasing compliance for screening. However, applying lubricating gel on the vagina is not encouraged by gynaecologic literature, also students and resident doctors in training are advised against lubricating the speculum while collecting sample due to the worry that it may interfere with cytology results of cervical smears often leading to inadequacy.^17,19^ However there is paucity of convincing evidence to prove that using lubricating gel can prevent proper cytological analysis.^23,24,25^

To the researcher’s best knowledge, only few studies are published worldwide ^19,20,30, 33-39^ and none was done in this environment. The goal of this research work therefore is to find out if lubrication during speculum insertion has effects on the adequacy of cervical cytology smears and to determine if it decreases pain and discomfort in women undergoing cervical cancer screening by means of cervical smears and also compare the proportion of women willing to come for repeat cervical smears in the future.

**CHAPTER 2**

**LITERATURE REVIEW**

In this chapter, similar and related literature to this study were reviewed under the following sub-headings:

**Screening Options for Cancer of the Cervix**

**Options of speculum lubrication**

Water soluble lubricant gels

Topical analgesic containing lubricant gels

**Speculum lubrication and adequacy of cervical smears**

Studies on effects of lubrication on adequacy of cervical smears for cervical cancer screening.

**Speculum lubrication for cervical screening and pain perception**

Studies on pain perception following lubrication for cervical smear collection during cervical cancer screening

**Speculum lubrication for cervical smears and willingless for repeat cervical cancer screening**

**S**tudies on barriers affecting willingness for repeat cervical cancer screening

**Conclusion of literature review**

2.1 S**EARCH STRATEGY**

The following search engines were used: PubMed, Google scholar, Research gate, Medline and Cochrane library. The keywords for the search were Pap smear, Papanicolaou smear, cervical cytology, speculum lubrication, gel, KY jelly, and Pap smear result adequacy. Advanced PubMed search yielded the following search terms: (Effect[All Fields] AND lubricating[All Fields] AND gel[All Fields] AND ("patient comfort"[MeSH Terms] OR ("patient"[All Fields] AND "comfort"[All Fields]) OR "patient comfort"[All Fields]) AND ("vagina"[MeSH Terms] OR "vagina"[All Fields] OR "vaginal"[All Fields]) AND ("surgical instruments"[MeSH Terms] OR ("surgical"[All Fields] AND "instruments"[All Fields]) OR "surgical instruments"[All Fields] OR "speculum"[All Fields]) AND ("physical examination"[MeSH Terms] OR ("physical"[All Fields] AND "examination"[All Fields]) OR "physical examination"[All Fields] OR "examination"[All Fields])) AND ("randomized controlled trial"[Publication Type] OR "randomized controlled trials as topic"[MeSH Terms] OR "randomized controlled trial"[All Fields] OR "randomized controlled trial"[All Fields])

Additional references not identified by the initial search were retrieved from the reference section of the articles gotten from the search.

**2**.**2 Screening Options for Cancer of the Cervix**

Human Papilloma Virus test, Papanicolaou test, colposcopy, visual inspection with the use of Acetic acid (VIA) then Visual Inspection with Lugol’s iodine (VILI) are methods presently used for cervical cancer screening.^26^ The aim is to identify people who have greater chances of developing the illness which are women with the premalignant lesions. Presently screening modality is subject to obtainable resources in the population to be screened.

Visual inspection is presently employed in poor income countries. VIA has 85 per cent specificity and 79 per cent sensitivity.^27^ In VILI sensitivity is improved by 10% but specificity remains unchanged. Although there are shortcomings with these methods, they may reduce cervical cancer rates in low income countries.^27^

Screening using the Papanicolaou test is a recognized modality of screening. A decline in both the occurrence and death from carcinoma of the cervix has been documented. Issues however has been raised concerning false negative rates of Pap smear.^28^ Measures to reduce the false negatives include liquid based preparations and quality control in cytology and colposcopy and repeat cytology.

Recently HPV testing has been introduced. HPV testing is efficient and reliable. High risk HPV is found in majority (99.8%) of cases of cervical cancer. It has high negative predictive value (90.6%) and high sensitivity (100%). ^26^

2.3 **Options of speculum lubrication**

The speculum can stick to the vulva and vagina and cause discomfort, pain or even tears during pelvic examinations. This can give rise to aversion for vaginal examination. In order to ensure patient comfort and satisfaction speculum lubrication has become pertinent.

Lukewarm water can be used for speculum lubrication. Water will reduce friction between the speculum and the vulva and vaginal walls and reduce discomfort to some extent. Water has no chemical component so this method has the least likelihood of affecting the quality of the cervical smear sample.^29^

Water soluble lubricant gels are other options for speculum lubrication and have been shown to be more effective than water. In the United States of America (USA), Hill et al in their study in 2012 randomized 120 women into two groups. One group had before insertion, application of 3mls of water to a plastic speculum and the other group had 0.3ml of gel applied to the speculum before insertion. The women were not aware of their intervention arm placement and were told to state their intensity of pain on a 0 to 10 visual analogue scale (VAS) following speculum opening. Significantly lesser pain was documented for women in the gel intervention arm compared to that of water. (Mean scores 1.4 versus 2.2) Also, more scores of ‘0’ were documented in the gel intervention arm. (34% versus 10%).^30^

Topical analgesic containing lubricant gels can also be used to lubricate the speculum during vaginal examinations. In 2012, Keskin et al demonstrated in their study in Turkey that topical analgesic containing lubricant gel is more effective than water soluble lubricant gel in reducing pain and discomfort during vaginal examinations. In this study the women were placed at random to an EMLA (lidocaine-prilocaine) arm, lubricating gel arm and a control arm. Using the VAS scale the women were told to state their level of pain at insertion, during opening and following removal of speculum. Women in the EMLA group recorded significantly lower pain values at all stages of speculum use than their counterparts in the lubricant gel arm and control arm (P<0.001) while women in the gel arm recorded lower pain values compared to their counterparts in the control group except in the second phase of the procedure. P<0.001. ^31^

It has also been documented that carbopol polymers containing lubricating gel may interfere with cervical screening tests. Lin et al in USA did a retrospective study of liquid based Pap smears obtained from January 2010 to March 2012 by the gynaecological oncology unit of a hospital. A sum of 2041 cervical and 1930 vaginal smears were obtained. Of these 675 cervical and 765 vaginal samples were obtained using lubricants while 1366 cervical and 1165 vaginal samples were obtained with no lubricant. Results showed no significant difference in adequacy of smears obtained without lubricating gel versus smears gotten with a lubricant devoid of carbomers. However, inadequate smear rate was significantly high in smears obtained with the use of lubricant gels containing carbomers and mostly noted for vaginal smears. (26.9% compared to 1.2%, OR =30.3, 95% CI =16.6-55.1, P<0.0001).^32^

**2**.**4 Speculum lubrication and adequacy of cervical smears**

The practice of speculum lubrication for vaginal examination is varied due to the fears that lubricants can alter the adequacy of cytological smears. However, recent studies have investigated the relationship between speculum lubrication and Pap smear accuracy and showed evidence supporting that speculum lubrication does not disrupt the evaluation of cytological smears.

Amies et al^33^ in their study randomized five public health family planning clinics in USA to lubricating gel and water arms. A sum of 2906 Pap smear specimens were obtained, 1440 from units using lubricants and 1466 from the non-lubricant using clinics. A dime size of the lubrication gel was stated to have been applied on the external part of the inferior blade of the plastic disposable speculum which is not objective as this might be difficult to ascertain by different people because a dime is not a standard way of measurement. Inadequate smear rate for the clinics using lubricant compared to clinics not using lubricants was 1.4% versus 1.3%. The percentage of inadequate smears for the clinics using gel did not differ significantly from those of the control clinics [Odds ratio (OR) 1.1; 95% Confidence Interval (CI) 0.6, 2.0]. Reasons for unsatisfactory smears were scanty cells, excess blood and inflammation. Drying artefact or gel overlay was not encountered as causes of encumbrance in evaluation. They did not evaluate the pain level during speculum insertion.^33^

In 2012, Uygur et al ^20^ demonstrated in Turkey that speculum lubrication did not alter cervical cytological smears. A total of 400 eligible women were grouped at random into two arms: the gel and no gel groups. Each arm was then subsequently randomly divided to have liquid based smears or conventional smears. Dime sized gel was spread on the outer side of the lower blade as done by Amies et al which is not a standard way of measurement and will not be easily reproducible. Out of the 400 women only 3 unsatisfactory results were recorded, one from the group that did not use gel( 0.5%) and two from the other group(1%).^20^

In a prospective randomized study in Turkey by Simavli et al ^34^ in 2013, samples of 1580 patients were evaluated. The lubricating gel was spread on the exterior side of the speculum. The rates of unsatisfactory cytopathologic examination were 1.13% and 1.39% respectively for the gel and no gel groups which did not differ significantly. (p<0.001). Simavli et al concluded that lubrication of speculum does not affect the quality of the Pap results. ^34^

In USA, Gilson et al ^19^ randomized 70 patients from July 2001 to May 2002. Thirty women were assigned at random to the no gel arm and 40 to the gel arm. All women recruited underwent two consecutive Pap smears. The first was performed using dry speculum and the second was done using speculum lubricated with 2.7g water soluble lubricant gel on the external surface. The two unsatisfactory smears from the gel arm were as a result of obscuring blood. Vaginal lubricant was not stated as a cause of unsatisfactory smear. The number of inadequate Pap smears (p=0.50) did not differ significantly in the two groups.^19^

In another study conducted in a clinic in USA by Griffith et al,^36^ eight consecutive months were randomly assigned to be gel speculum lubrication months or water moistened speculum months. Lubricating gel of about the size of a dime was applied on the distal end of the speculum. The patients either had Pap smear alone or combination DNA probe assay for Neisseria gonorrhoeae and Chlamydia trachomatis or both. Out of the 6538 patients evaluated from July 2003 to February 2004, 3460 had Pap smears while 5535 had combination probe assays for Neisseria gonorrhoeae and Chlamydia trachomatis. Results showed that the rate of unsatisfactory smears was 1.1% during the period gel was used versus 1.5% when water was used [Odds Ratio (OR) 0.74; 95% Confidence Interval (CI) 0.41–1.35]. The rate of detection of endocervical Chlamydia trachomatis was the same [1.5%] (OR 1.05; 95% CI 0.67–1.62) during the period gel was used and when water was used. They concluded that metal speculum lubrication with a little amount of lubricant compared to water did not raise the rate of unsatisfactory smear result or reduce the rate of detection of Chlamydia trachomatis. ^36^

A meta- analysis by Pergialiotis et al ^23^ in 2015 evaluated five randomized controlled studies and two quasi randomized controlled studies involving 8717 women of whom 4450 (51%) had speculum lubrication and 4267 (49%) made up the control arm. Results showed no significant variation in the unsatisfactory results rates in the control versus lubrication groups.(8398 women, REM, OR =0.94, 95% CI = 0.64-1.37). Also pain scores did not differ significantly in both groups. They concluded that speculum lubrication does not alter conventional cytological evaluation of cervical smears. ^23^

However, Charoenkwan et al ^38^ studied a total of 1334 patients in Thailand between July 2005 and April 2006 and concluded that contamination with lubrication gel can negatively alter the accuracy of cervical smears. Two consecutive cervical smears were collected from the women. They took the first smear (uncontaminated) through the routine collection technique. Then 1 to 1.5cm of lubricant was applied on the cervix before taking the second smear. Inconsistency in results between the two samples and smear adequacy were evaluated. There were statistically significantly greater numbers of unsatisfactory smears in the samples that were contaminated with gel.(12.1% vs. 1.7%) P < 0.01. Among the satisfactory smears, the inconsistency in results between the smear contaminated with gel and the smear not contaminated with gel from the same individual was 0.3%. ^38^

Similarly, Kosus et al in 2012 studied a total of 1,999 patients. The women were allocated into two arms. In the control group, smears were collected with a dry speculum while the speculum was lubricated in the gel group. After evaluation results showed rate of unsatisfactory samples to be 2.2%. In the gel group, the rate of unsatisfactory smear wash statistically significantly higher. The risk of inadequate results was further raised by samples collected by residents.^39^

**2.5 Speculum lubrication for cervical smears and pain perception**

In the study stated above by Uygur et al ^20^ Numeric rating scale was employed also to evaluate pain at the end of the procedure. In the arm that did not use gel, pain scores were significantly greater compared to the arm that used gel (mean 2.3 [95% CI, 0-8] and mean 1.6 [95% CI, 0-7], respectively P<0.05). ^20^

Simavli et al ^34^ also demonstrated in their study above that speculum lubrication significantly reduced pain during Pap test. Numeric pain scale was employed to assess pain during the introduction and opening of the speculum. Pain scores were less in the gel arm compared to the non- gel group with mean scores of 3.0 and 4.0 respectively (mean difference -1.00, IV fixed-1.20, 95% CI 0.80)^34^

Gilson et al^19^ in their study above however reported that speculum lubrication did not affect pain and discomfort. All women were told to score the pain of each Pap smear on a Wong-Baker Faces Pain Rating Scale and the discomfort level rating (p=0.69) did not differ significantly in the two groups. ^19^

Gungorduk et al^35^ in a randomized controlled trial in Turkey in 2015 analysed 200 women who had speculum examination. One hundred women each were assigned to the lubricating gel and water arms. They applied 6.5ml of lubricating gel to the external surfaces and distal tips of the speculum. Their pain levels were documented using the VAS. The pain levels recorded at all stages of the speculum examination were statistically significantly less in the lubricating gel arm compared to the arm that used water at introduction (3.95 ± 1.57 vs. 5.28 ± 1.71, P < 0 t.001), opening (5.96 ± 1.48 vs. 6.74 ± 1.69, P < 0.001) and removal stages (2.60 ± 1.17 vs. 3.50 ± 1.25 P < 0.001). The authors concluded that speculum lubrication significantly reduced pain caused by speculum examination among gynae-oncology patients. ^35^

Another meta- analysis by Bakker et al ^37^ in 2017 included five studies with a total of 2383 subjects. A sum of 1232 women had speculum lubrication while 1221 women had no speculum lubrication. Discomfort was recorded at several stages in the procedure and the authors reported statistically significant decline with introduction (mean difference -0.98;95% CI =-1.13 to -0.83, 5 studies, 2383 subjects), dilatation (mean difference, -1.52; 95% CI= -2.43 to -0.61, 3 studies, 1864 subjects) and removal (mean difference -0.97; 95% CI= -1.29 to -0.65, 2 studies, 284 subjects). The authors concluded that speculum lubrication with water soluble lubricant consistently decreased discomfort across each population. ^37^

**2.6 Speculum lubrication and willingness for repeat cervical cancer screening**

Whether or not speculum lubrication will affect willingless for repeat cervical cancer screening in women that received speculum lubriction and no speculum lubrication in their initial cervical cancer screening has never been tested.

Speculum lubrication may result in more individuals accepting to participate in repeat cervical cancer screening, because it may remove some of the barriers such as pain and discomfort that prevent women from participating in regular screening programs. Several studies have shown that the majority of women’s experience of discomfort or pain at a past clinical visit usually discourage women from visiting a health professional again for repeat cervical cancer screening.^43,44,45^

In a systematic review on experiences of cervical screening and barriers to participation in the context of an organised programme by Chorley et al,^43^ the cervical screening was described as a highly embodied experience. In the Chorley et al review, some women described the procedure for cervical smear collection as uncomfortable or even painful and reported side effects of the test, including lasting pain and bleeding. Additionally, Chorley et al systematic review revealed that women also commented on two aspects of the procedure, which they found particularly unpleasant: the taking of the sample and, in particular, the speculum, and its coldness and the act of penetration. In this study, previous screening experiences seemed to influence decisions regarding re-attendance in two ways. Firstly, previous normal results were sometimes interpreted as being given an ‘all clear’ with no further need to attend. Conversely, one woman reported how a previous positive result reduced her desire to attend screening because of the increased anxiety that she now felt. Secondly, even when screening was still considered relevant, previous experiences influenced willingness to re-attend. Chorley et al review further stated that for some women, a single negative experience prevented them from re-attending screening, even if they had multiple positive previous experiences to draw upon. This studies involved in this systematic review was not a randomised controlled trials and did not compare the failure at recreening in women that received speculum lubriction versus no speculum lubrication. ^43^

 In Jia et al study,^45^ anxious feeling once the disease was diagnosed (47.6%), no symptoms/discomfort (34.1%) and do not know the benefits of cervical cancer screening (13.4%) were the top three reasons for refusing cervical cancer rescreening. Additionally, Jia et al study revealed that women who were younger than 45 years old or who had lower incomes, positive family histories of cancer, secondary or higher levels of education, higher levels of knowledge and fewer barriers to screening were more willing to participate in cervical cancer screenings than women without these characteristics. This study was not a randomized controlled trial and did not compare the failure at recreening in women that received speculum lubriction versus no speculum lubrication. ^45^

**2.7 CONCLUSION OF LITERATURE REVIEW**

The reviewed studies had some limitations, ranging from the fact that some of the studies were not randomized controlled studies, mainly conventional Pap smear techniques were utilized and all the studies were conducted in high income countries where the cervical cancer screening facilities and uptake are more prevalent. In addition none of the studies reviewed compared the proportion of women willing to come for repeat cervical smears in the future after undergoing initial cervical cancer screening with speculum lubrication and without speculum lubrication.

Some of the studies were not randomized control trials, ^44,45^ and in some the randomization was for the clinics and months of study and not direct patient randomization.^33,36^ The presently proposed study will be a randomized control study.

The amount of lubricants varied, in some studies a dime sized gel was used ^20,33^ and in others 0.3 to 6.5ml of lubricant gel was used. ^30,35^The site of application also differed, in some works the gel was spread on the entire external surfaces of the speculum^19,34^ while some researchers applied gel to the tip or distal end of the speculum^30,36^ and direct lubrication of the vaginal canal and introitus was employed in a study.^31^ In one of these studies, the lubricating gel was applied directly on the cervix ^38^and may have led to higher rates of unsatisfactory Pap smear results. This shows that the quantity of lubricant that got into the vagina varied and hence may result in different outcomes among the studies. 3mls of gel will be employed in this study and applied on the entire external surface of the speculum for reproducibility.

The Numeric rating,^20,34^ Wong-Baker Faces pain rating ^19^ and Visual analogue scales ^30,35^ were methods used to assess pain in the various studies reviewed. Numeric rating scale for pain will be used in the presently proposed study because it is authenticated and shown to be widely appropriate for pain evaluation.^20^

This proposed study will address these limitations and fill the research gap by adopting a randomized control approach and employing liquid-based cytology smear techniques to determine if speculum lubrication has effects on the adequacy of cervical cytology smears and to determine if it decreases pain and discomfort in women undergoing cervical cancer screening in low income country of Nigeria and further evaluate the proportion of women willing to come for repeat testing in the two groups.

**CHAPTER THREE**

**AIM AND OBJECTIVES**

**3.1 AIM**

To determine the effects of speculum lubrication on perceived pain and the adequacy of cytological smear results during Pap smear examination at Nnamdi Azikiwe University Teaching Hospital (NAUTH) Nnewi, Anambra State, Nigeria.

**3.2 SPECIFIC OBJECTIVES**

1. To compare the pain experienced by women undergoing cervical cancer screening with speculum lubrication and those without speculum lubrication.

2. To compare the proportion of unsatisfactory cytology smear results of women undergoing cervical cancer screening with speculum lubrication and those without speculum lubrication.

3. To compare the proportion of women willing to come for repeat cervical smears in the future

after undergoing initial cervical cancer screening with speculum lubrication and without speculum lubrication.

3.3 **RESEARCH QUESTIONS**

1. Does speculum lubrication affect the comfort of women undergoing cervical cancer screening using Pap smear?

2. Does speculum lubrication affect the adequacy of cytology results of women undergoing cervical cancer screening using Pap smear?

3.**4 Null hypothesis**

1. Speculum lubrication does not significantly affect the comfort of women undergoing cervical cancer screening using Pap smear.

2. Speculum lubrication does not significantly affect the adequacy of cytology smears of women undergoing cervical cancer screening using Pap smear.

**3.5 Alternative hypothesis**

1. Speculum lubrication significantly affects the comfort of women undergoing cervical screening using Pap smear.

2. Speculum lubrication significantly affects adequacy of cytology smears of women undergoing cervical screening using Pap smear.

**CHAPTER FOUR**

**RESEARCH METHODOLOGY**

4.**1 STUDY DESIGN**

The work will be a randomized controlled study.

4.2 **STUDY SETTING**

This work will be done in the gynaecology clinic of Nnamdi Azikiwe University Teaching Hospital (NAUTH) Nnewi, Anambra State Nigeria. NAUTH is the only federal teaching hospital in Anambra State, a state in the South eastern part of the country. It offers comprehensive and tertiary services to occupants of Anambra, Enugu, Delta, Imo and Abia states. It is structured to offer medical undergraduate and medical postgraduate training as well as paramedical training. It is also well grounded in medical research. This teaching hospital is well equipped to offer obstetric, gynaecological and gynae-oncology services both on in-patient and outpatient bases. Nnewi is one of the biggest towns in Anambra state and consists of 4 villages which include Nnewichi, Uruagu, Umudim, and Otolo. ^41^ It is a place known for its industrial and commercial activities. It is home to the popular Nkwo Nnewi market, the biggest market for motorcycle spare parts in West Africa.^41^ It is home also to major automobile, petrochemical and cosmetic industries and the first indigenous car producing plant in Nigeria. The inhabitants are mainly Christians who speak Igbo majorly and are mostly traders.

4.**3 SAMPLE SIZE DETERMINATIONf**

The sample size will be calculated using the formula for sample size Determination of minimum sample size for experiemtal studies (Difference in proportions) ^42^ substituting for the values of proportion of unsatisfactory smears as found in a previous study ^20^

n **=** [(Z**_β_** + Z**_α/2_**)^2^ x 2P(1−P)] **=** Minimum sample size

E ^2^

Where

P **=** (P_1_ + P_2_) / 2 **=** (0.01+ 0.005) / 2 **=** 0.0075

E **=** Effect size **=** P_1_ - P_2_ **=** 0.01- 0.005 **=** 0.005

Z**_β_ =** Corresponding Z value at 80% Power **=** 0.84

Z**_α/2_** **=** Corresponding Z value at 95% confidence level **=** 1.96

n **=** [(0.84 + 1.96)^2^ x 2(0.0075)(0.9925)] **=** 0.116718 **=** 4668.72 **≈** 4669

(0.005) ^2^  (0.005) ^2^

Adjusting for finite population, N = 60 samples from clinic in 3 months.

n_s_ **=** n^1^ / 1 + n^1^/N

Where;

n_s_ **=** Adjusted sample size

n^1^ **=** Calculated sample size

N **=** population for study

n_s_ **=** 4669 / (1 + 4669/60) = 4669/78.82 = 59.2 **≈** 60

This will be approximately 60 making a total of 120 with 60 controls and the other 60 for intervention arm.

However, considering attrition rate of 10.0% will give 6 subjects, there will be 6 women in each of intervention and control groups. Therefore, the total number of subjects in each arm = 60 + 6 = 66. So a total of 132 subjects will be recruited for the study.

4.4 **ETHICAL CONSIDERATIONS**

Ethical approval will be gotten from the Ethics Committee of Nnamdi Azikiwe University Teaching Hospital Nnewi before embarking on the study. An informed consent will be gotten from patients before they are selected to participate in the research. The women will choose to take part in the research or withdraw at any time and will be assured of getting standard quality of care without prejudice. The researcher will fund the research and none of the participants will pay for the procedure or investigation. There will be minimal discomfort to the participants. The research assistants and participants will not be given incentives. Information gathered from this study will be treated as strictly confidential.

4.5 **STUDY POPULATION**

The women will be recruited from consenting patients who are at least 25years old who present to the gynaecological clinic of NAUTH Nnewi for routine Pap smear.

4.6 **INCLUSION CRITERIA**

The participants will include pre and post-menopausal women who require Pap smear.

4.7 **EXCLUSION CRITERIA**

The following women would be excluded from the work virgins, pregnant women, women having their menstrual period, women with any overt cervical pathology, women with vulvar pathologies or those on hormone replacement therapy. Women with vaginitis, those undergoing vulvectomy or vaginectomy and women who had fertility-sparing surgery will not be recruited.

4.8 **DATA COLLECTION**

Training will be organized for five Senior Registrars who will work as research assistants. The researcher and the 5 trained research assistants will collect data from the participants at the gynaecological clinic with the aid of a proforma. NAUTH runs outpatient services on every working day and emergency services for 24 hours every day. On the average 2 smears are collected each day for cervical cancer screening in the gynaecological clinic making 10 in a week and 40 samples in a month. So it is expected that the sample size of 102 will be achieved in 3 months.

4.**9 SAMPLING APPROACH, RANDOMIZATION AND ALLOCATION SEQUENCE**

Purposive sampling approach will be employed. Following informed consent, selected women will be assigned at random into two arms, namely the gel group and no gel group with the aid of computer created sporadic numbers. The sequence of randomization will be generated by tfhe computer via randomly permuted blocks (blocks of 4, allocation ratio 1:1). An independent person will perform the randomization and will not be involved in the study. The independent person will create a randomization table using computer software program available at <http://mahmoodsaghaei.tripod.com/Softwares/randalloc.html>. Serially numbered opaque sealed envelopes will be used for allocation concealment. The study interventions would be printed in a sheet of paper put in serially numbered opaque closed envelopes. The envelopes would be kept and opened by an independent person. Once the envelope is opened the allocation of the participant would not be changed. Each of the envelopes will contain either a sheet of paper showing gel group (Group A) placing the patient into the group using the speculum lubrication or a paper displaying no gel group (group B) placing the patient into the group receiving dry speculum (without speculum lubrication). Participants cannot be switched from one group to the other once they are placed in a group.

4.10 **STUDY PROCEDURE**

The consent for Pap smear will be gotten for each person during the visit. After explaining the procedure, the woman will be placed in dorsal position with the legs flexed at the knee and adducted at the hips.

Latex gloves will be worn by the researcher or trained research assistants and 2mls of KY jelly (Dionel, Maryland -USA) will be applied on the entire external surfaces of the metal Cusco’s speculum and inserted gently into the vagina to reveal the cervix in women assigned to the gel group. Appropriate size of Cusco’s speculum will be determined for each participant. A dry speculum (no lubricant) will be used for the control group. In all women, speculum insertion will be done as follows: first, the labia majora and minora will be parted to put in the speculum, which will be introduced at an angle of 45-degrees facing downward and then horizontally rotated. After full insertion, the bills of the speculum will be opened until the vaginal cuff is seen and the speculum is fastened by screwing of the thumbnut. A cotton tipped swab will be used to clear any mucous on the cervix. A cytobrush (Diapath S.p.A Martinego, Italy) will be used to take the sample for Pap smear, after obtaining the sample, the head of the brush is removed and put into the liquid preparative collection vial containing a liquid based medium. (Diapath S.p.A Martinego, Italy) The specimen in the vial will then be carefully mixed in the liquid based medium. Then, the speculum will be gently withdrawn. The patient will be cleaned and counselled on the outcome. In addition, after each smear the patient will be asked to rate her discomfort at the end of the procedure on the Numeric Rating Scale for pain with numbers starting from 0 (no discomfort) to 10 (most discomfort). The patient will also state if she will be willing to come for repeat testing in the future. Numeric Rating Scale for pain will be used because it is authenticated and is widely accepted to be appropriate for evaluation of pain. ^20^

The vial will then be taken to the histopathology laboratory of Nnamdi Azikiwe University Teaching Hospital, Nnewi where it would be transferred into a centrifuge tube with same quantity of cleaning solution. The fluid will then be centrifuged for 10 minutes at 1400 rpm; the supernatant will be removed. Smears will be made from the sediments after mixing with the cellular base solution (Diapath S.p.A Martinego, Italy) and stained by the Papanicolaou staining method and reported using the Bethesda system stating whether the sample is satisfactory or not and the reason for unsatisfactory results. Samples will be recorded as “unsatisfactory” if no endocervical cells are present, if they are obscured by blood, or if drying artifact or gel overlay are present; otherwise, they will be recorded as “satisfactory”. ^19^ The histopathologist will not be aware of the intervention arm (group of patients) to which the smears to be analysed belong.

4.11 **OUTCOME MEASURES**

The primary outcome measures will be the mean NRS pain scores and proportion of women with inadequate cervical cytology smears while the secondary outcome measures will be the pattern of Pap smear results and proportion of women willing to come for repeat testing.

4.12 **PLANNED HANDLING OF RESULTS**

4.12.1 DATA AND STATISTICAL ANALYSIS

Statistical Packages for Social Sciences (SPSS) version 25 would be employed in the analysis of results. Charts and tables would be used to represent collected data. Means and standard deviations will be used to represent continuous data. T-test would aid the assessment of parametric variables and Mann Whitney U-test would be used for non-parametric ones. Statistical test of significance would be deduced at p-value less 0.05.

DUMMY TABLE 1 SOCIO-DEMOGRAPHIC AND CLINICAL CHARACTERISTICS OF THE STUDY POPULATION

| CHARACTERISTIC | ALL | GEL GROUP | NON GEL- GROUP | P-VALUE |
| --- | --- | --- | --- | --- |
| AGE: (years)  25-30  31-40  41-50  Above 50 |  |  |  |  |
| PARITY:  P0  P1  P2-4  P>5 |  |  |  |  |
| MARITAL STATUS  Single  Married  Divorced  Widowed |  |  |  |  |
| MENOPAUSAL STATUS:  Pre-menopausal  Post-menopausal |  |  |  |  |
| USE OF HORMONAL DRUGS OR DEVICES:  Positive history  Negative history |  |  |  |  |

TABLE 2. PAP SMEAR SAMPLE ADEQUACY

| **Laboratory Findings** | **All** | **Gel** | **No gel** | **P-value** |
| --- | --- | --- | --- | --- |
| Satisfactory Pap smear sample |  |  |  |  |
| Unsatisfactory Pap smear sample |  |  |  |  |

TABLE 3. PAP SMEAR RESULT PATTERNS

| Pap smear results | All | Gel | No gel |
| --- | --- | --- | --- |
| HSIL |  |  |  |
| LSIL |  |  |  |
| ASC-US |  |  |  |
| ASC –H |  |  |  |
| BCC |  |  |  |
| WITHIN NORMAL LIMITS |  |  |  |

**List of abbreviations:** HSIL-High Grade Squamous Intraepithelial Lesion, LSIL- Low Grade Squamous Intraepithelial Lesion, ASC-US- Atypical Squamous Cells of Undetermined Significance. ASC-H- Atypical Squamous Cells in which a High-Grade Squamous Intraepithelial Lesion (HSIL) cannot be excluded, BCC-Benign Cellular Changes.

**TABLE 4: WILLINGNESS TO COME FOR REPEAT TESTING**

| Willingness to come for repeat testing | ALL | GEL | NO GEL | P VALUE |
| --- | --- | --- | --- | --- |
| YES |  |  |  |  |
| NO |  |  |  |  |

FIGURE 1. NUMERICAL RATING SCALE FOR PAIN

4.13 **STRENGTH AND LIMITATIONS OF THE STUDY**

This study is the first of its kind in a low income country like Nigeria, The limitation is that only one type of water- based lubricant will be used so results may not be applied to other kinds of lubricants. Perception of pain is complex and multifactorial. Cultural and genetic factors can affect these results in different populations.

Pap smear collection is provider technique dependent. Even though the researcher and research assistants will all be senior resident doctors with same level of training and experience, there still may be chances of inter provider differences. Except for the histopathologist, the researcher, research assistants and patients will be aware of the intervention assignment because given the nature of the intervention technique, blinding of participants and researcher/ research assistants will be difficult.

**4.14 CONFLICT OF INTEREST**

There would be no conflict of interests.

**APPENDIX 1**

**CONSENT FORM TO PARTICIPATE IN A RESEARCH STUDY**

This form contains all the required details which you need to enable you choose whether to participate or not in this work titled:

**EFFECTS OF SPECULUM LUBRICATION ON CERVICAL SMEARS FOR CERVICAL CANCER SCREENING: A DOUBLE-BLIND RANDOMIZED CONTROLLED TRIAL**

This work is to be done by DR ILIKA CHITO PACHELLA, a Senior Registrar in the department of Obstetrics and Gynaecology in Nnamdi Azikiwe University Teaching Hospital, Nnewi.

**STUDY PURPOSE**

By this consent form, you are asked to participate in this work. Your resolution to be a part of this study is entirely your choice. You will not be refused care if you choose not to be involved. You are free to pull out at any point you want. You will not pay for the procedure and you will not receive any payment for being a part of this work.

Cancer of the tip of the womb is the commonest cancer of the genital organs of women occurring in our environment. Early detection and prompt treatment will help to eradicate this disease. Pap smear is an effective method of screening for the disease but due to pain and discomfort during Pap smear test, most women do not present for Pap smear or a repeat of the test. Speculum lubrication may reduce pain and discomfort during specimen collection for Pap smear test but it may also reduce the detection rate of an abnormality while taking specimen for Pap smear.

The reason for this study is to find out if speculum lubrication affects the correctness of the Pap test and whether it decreases pain while collecting Pap smear specimen

**STUDY PROCEDURE**

The study will involve women presenting for routine Pap smear. If you resolve to be a part of this work, a Pap smear will be collected from you. Using a clean stick, cells from the tip of the womb will be taken, smeared on a slide and taken to the laboratory for analysis. After that you will be asked to state how painful the procedure was using a scale of 0 to 10.

**STUDY RISKS**

There are no severe risks involved in taking part in this work. You may have slight pain or discomfort during the procedure but it will be done gently.

**POTENTIAL BENEFITS**

The benefit of participating in this study is that the cost of the Pap smear screening will be paid by the researcher.

**VOLUNTARY PARTICIPATION**

You can resolve to be a part of this work or not. You will not be refused appropriate medical care for your case whether you are involved in the work or not. You are free to pull out at any point and it would not interfere with the management you are getting.

**CONFIDENTIALITY**

Details obtained in this work will be kept confidential.

**QUESTIONS**

Do not hesitate to ask questions at any time.

I hereby decide to be involved in the proposed work as I have been informed in English or Igbo language and understood by me.

It is clear to me that being a part of this work is by choice and if I pull out of the work, I would still receive the exact quality of treatment offered to other women by the caregiver.

PARTICIPANT’S SIGNATURE OR THUMBPRINT….………….…DATE…………………

SIGNATURE OF WITNESS…………………………………………...DATE….………………

RESEARCHER - **DR ILIKA CHITO PACHELLA**

RESEARCHER’S PHONE NUMBER—**08032017499**

**APPENDIX 2**

**RESEARCH PROFORMA**

**EFFECTS OF SPECULUM LUBRICATION ON CERVICAL SMEARS FOR CERVICAL CANCER SCREENING: A DOUBLE-BLIND RANDOMIZED CONTROLLED TRIAL**

Please do fill the parameters below. Tick [X] where appropriate.

Code No………Folder Number………………Date of Enrolment……………

**PARTICIPANT INFORMATION SECTION**

1. **ELIGIBILITY CHECK LIST**: A subject is not eligible if the answer to any of these is YES
2. Menstruating YES [ ] NO [ ]
3. Pregnant YES [ ] NO[ ]
4. Cervical cancer patient YES [ ] NO [ ]
5. Vulvar lesion or malignancy YES [ ] NO[ ]
6. History of cervical or vulvar surgeries YES [ ] NO[ ]
7. Hormone therapy YES [ ] NO[ ]
8. Vaginitis YES [ ] NO[ ]

1. **BIODATA**
2. **AGE AS AT LAST BIRTHDAY(YEARS)…**……25-30 [ ] 31-40 [ ] 41-50 [ ] >50 [ ]
3. **PARITY**:

P0 [ ] P1 [ ] P2 [ ] P3 [ ] P4 [ ] P5 [ ] P>5 [ ]

1. **MARITAL STATUS:**

SINGLE [ ] MARRIED [ ] DIVORCED/SEPARATED [ ] WIDOWED [ ]

1. **EDUCATIONAL STATUS:**

NO EDUCATION [ ] PRIMARY [ ] SECONDARY [ ] TERTIARY [ ]

1. **RELIGION:**

CHRISTIANITY [ ] ISLAM [ ] TRADITIONAL [ ]

1. **OCCUPATION………………………………………**
2. **ETHNICITY**

IGBO [ ] HAUSA [ ] YORUBA [ ] OTHERS Specify……

1. **SMEAR SAMPLE STATUS**

SATISFACTORY [ ] UNSATISFACTORY [ ]

UNSATISFACTORY( SPECIFY REASON)………………

1. **PAP SMEAR RESULT**

HSIL [ ] LSIL [ ] ASCUS [ ] ASC-H[ ] BCC [ ] NORMAL LIMIT[ ] INFECTION/CERVICITIS [ ]

1. **NUMERIC RATING SCALE-** what number best describes your pain during the procedure

**0 [ ] 1[ ]2 [ ] 3 [ ]4[ ]5 [ ] 6 [ ]7 [ ] 8 [ ] 9[ ]10 [ ]**

**(where 0 is No pain and 10 worst possible pain)**

1. **SATISFIED WITH PAP SMEAR PROCEDURE**

YES [ ] NO [ ]

1. **ARE YOU WILLING TO COME FOR REPEAT TESTING IN THE FUTURE**

YES [ ] NO [ ]

If no give reasons……………………………………………………………………..

**REFERENCES**

1. [Ferlay J](https://www.ncbi.nlm.nih.gov/pubmed/?term=Ferlay%20J%5BAuthor%5D&cauthor=true&cauthor_uid=30350310), [Colombet M](https://www.ncbi.nlm.nih.gov/pubmed/?term=Colombet%20M%5BAuthor%5D&cauthor=true&cauthor_uid=30350310), [Soerjomataram I](https://www.ncbi.nlm.nih.gov/pubmed/?term=Soerjomataram%20I%5BAuthor%5D&cauthor=true&cauthor_uid=30350310), [Mathers C](https://www.ncbi.nlm.nih.gov/pubmed/?term=Mathers%20C%5BAuthor%5D&cauthor=true&cauthor_uid=30350310), [Parkin DM](https://www.ncbi.nlm.nih.gov/pubmed/?term=Parkin%20DM%5BAuthor%5D&cauthor=true&cauthor_uid=30350310). Estimating the global cancer incidence and mortality in 2018: GLOBOCAN sources and methods. [Int J cancer.](https://www.ncbi.nlm.nih.gov/pubmed/30350310) 2019 Apr 15; 144 (8):1941-1953.
2. [Eleje GU](https://www.ncbi.nlm.nih.gov/pubmed/?term=Eleje%20GU%5BAuthor%5D&cauthor=true&cauthor_uid=30888060), [Eke AC](https://www.ncbi.nlm.nih.gov/pubmed/?term=Eke%20AC%5BAuthor%5D&cauthor=true&cauthor_uid=30888060), [Igberase GO](https://www.ncbi.nlm.nih.gov/pubmed/?term=Igberase%20GO%5BAuthor%5D&cauthor=true&cauthor_uid=30888060), [Igwegbe AO](https://www.ncbi.nlm.nih.gov/pubmed/?term=Igwegbe%20AO%5BAuthor%5D&cauthor=true&cauthor_uid=30888060), [Eleje LI](https://www.ncbi.nlm.nih.gov/pubmed/?term=Eleje%20LI%5BAuthor%5D&cauthor=true&cauthor_uid=30888060). Palliative interventions for controlling vaginal bleeding in advanced cervical cancer. [Cochrane Database Syst Rev.](https://www.ncbi.nlm.nih.gov/pubmed/30888060) 2019 Mar 19; 3: CD011000.
3. Ikechebelu JI, Onyiaorah IV, Ugboaja JO, Anyiam DCD, Eleje GU. Clinicopathological analysis of cervical cancer seen in a tertiary health facility in Nnewi, South‐east Nigeria. J Obstet Gynaecol. 2010; 30(3):299-301.
4. Louie KS, de Sanjose S, Mayaud P. Epidemiology and prevention of human papilloma virus and cancer in sub-Saharan African: a comprehensive review. Tropical Med Int Health. 2009; 14:1287–1302.
5. Parkin DM, Sitas F, Chirenje M, Stein L, Abratt R, Wabinga H. Cancer in indigenous Africans-burden, distribution and trends. Lancet Oncol. 2008; 9:683–692.
6. Morounke SG, Ayorinde JB, Benedict AO, Adedayo FF, Adewale FO. Epidemiology and incidence of common cancers in Nigeria. J Cancer. Biol Res. 2017; 5 (3):1105.
7. Ahmed SA, Sabitu K, Idris SH, Ahmed R. Knowledge, attitude and practice of cervical cancer screening among market women in Zaria, Nigeria. Niger Med J. 2013; 54:316–9.
8. Awodele O, Adeyomoye AA, Awodele DF, Fayankinnu VB, Dolapo DC. Cancer distribution pattern in South-Western Nigeria. Tanzan J Health Res. 2011;13(2):125–31
9. Bruni L, Albero G, Serrano B, Mena M, Gómez D, Muñoz J, Bosch FX, de Sanjosé S. ICO/IARC Information Centre on HPV and Cancer (HPV Information Centre). Human Papillomavirus and Related Diseases in Nigeria. Summary Report 10 December 2018. Accessed on 2nd July, 2019.
10. Mwaka AD, Orach CG, Were EM, Lyratzopoulos G, Wabinga H, Roland M. Awareness of cervical cancer risk factors and symptoms: cross-sectional community survey in post-conflict Northern Uganda. Health Expect. 2015; 19(4):854–867.
11. Mittal S, Kamath AJ. Cytological screening for cervical cancer in women of reproductive age group. Int J Reprod Contracept Obstet Gynecol 2017; 6:5464-8.
12. Committee Opinion No. 704 Summary: human papillomavirus vaccination. Obstet Gynecol. 2017; 129(6):1155–1156.
13. Cox JT. Human papillomavirus vaccinations. In: Post TW, editor. UpToDate. Waltham, MA: [Accessed September 15, 2018].
14. White MC, Wong FL. Preventing premature deaths from breast and cervical cancers among underserved women in the United States: insights gained from a national cancer screening program. Cancer Causes Control, 2015; 26(5):805–9.
15. Miller JW, Royalty J, Henley J, White A, Richardson LC. Breast and cervical cancers diagnosed and stage at diagnosis among women served through the National Breast and Cervical Cancer Early Detection Program. Cancer Causes Control, 2015; 26(5):741–7.
16. Ekwueme DU, Uzunangelov VJ, Hoerger TJ, Miller JW, Saraiya M, Benard VB. Impact of the National Breast and Cervical Cancer Early Detection Program on cervical cancer mortality among uninsured low-income women in the U.S., 1991–2007. Am J Prev Med. 2014; 47 (3):300–8.
17. Hathaway JK, Pathak PK, Maney R. Is liquid based Pap testing affected by water based lubricant? Obstet Gynecol 2006; 107; 66-70.
18. Campos NG, Tsu V, Jeronimo J, Mvundura M, Kim JJ. Evidence-based policy choices for efficient and equitable cervical cancer screening programs in low-resource settings. Cancer Med. 2017; 6(8):2008–2014
19. Gilson M, Desai A, Cardoza-Favarato G, Vroman P, Thornton JA. Does gel affect cytology or comfort in the screening Papanicolaou smear? J Am Board Fam Med 2006; 19:340–4.
20. Uygur D, Guler T ,Yayci E. Association of speculum lubrication with pain and Papanicolaou test accuracy. J Am Board Fam Med 2012; 25:798-804.
21. Asiedu MN, Agudogo J, Krieger MS, et al. Design and preliminary analysis of a vaginal inserter for speculum-free cervical cancer screening. PLoS One. 2017; 12(5):e0177782. Published 2017 May 31.
22. Wright D, Fenwick J, Stephenson P, Monterosso L. Speculum 'self-insertion': a pilot study. J Clin Nurs, 2005; 14(9): 1098–1111.
23. [Pergialiotis V](https://www.ncbi.nlm.nih.gov/pubmed/?term=Pergialiotis%20V%5BAuthor%5D&cauthor=true&cauthor_uid=24769651), [Vlachos DG](https://www.ncbi.nlm.nih.gov/pubmed/?term=Vlachos%20DG%5BAuthor%5D&cauthor=true&cauthor_uid=24769651), [Rodolakis A](https://www.ncbi.nlm.nih.gov/pubmed/?term=Rodolakis%20A%5BAuthor%5D&cauthor=true&cauthor_uid=24769651), [Thomakos N](https://www.ncbi.nlm.nih.gov/pubmed/?term=Thomakos%20N%5BAuthor%5D&cauthor=true&cauthor_uid=24769651). The effect of vaginal lubrication on unsatisfactory results of cervical smears. [J Low Genit Tract Dis.](https://www.ncbi.nlm.nih.gov/pubmed/24769651) 2015 Jan; 19(1):55-61.
24. Allan GM, Korownyk C, Ivers N. Papanicolaou tests: does lubricant reduce the quality or adequacy? Can Fam Physician 2011; **57**:309.
25. Harmanli O,Jones KA. Using lubricant for speculum insertion. Obstet Gynecol 2010;116(2 Pt 1):415–7.g
26. Gravitt PE, Paul P, Katki HA. Effectiveness of VIA, Pap, and HPV DNA testing in a cervical cancer screening program in a peri-urban community in Andhra Pradesh, India. PLoS One. 2010; 5(10):e13711**.**
27. Arbyn M, Sankaranarayanan R, Muwonge R et al Pooled analysis of the accuracy of five cervical cancer screening tests assessed in eleven studies in Africa and India. Int J Cancer.2008 ;123(1):153-60
28. Koliopoulos G, Nyaga VN, Santesso N. Cytology versus HPV testing for cervical cancer screening in the general population. Cochrane Database Syst Rev. 2017; 8(8):CD008587.
29. Darvey DD, Cox JT Austin RM. Cervical cytology specimen adequacy: patient management guidelines and optimizing specimen collection**.** J Low Genit Tract Dis 2008; 12 (2):71-81.
30. Hill DA, Lamvu G. Effect of lubricating gel on patient comfort during vaginal speculum examination: a randomized controlled trial. Obstet Gynecol 2012;119(2 pt 1):227-31.
31. Keskin AE, Onaran Y, Duvan IC, Simavli S. Topical anaesthetic (lidocaine-prilocaine) cream application before speculum examination in post-menopausal women. J Minim Invasive Gynecol 2012; 19:350-5.
32. Lin S, Taylor J, Alperstein S, Hoda. Does speculum lubricant affect liquid‐based Papanicolaou test adequacy? Cancer (Cancer Cytopathol) 2014; 122:221–226.
33. Amies AM, Miller L, Lee SK, Koutsky L. The effect of vaginal speculum lubrication on the rate of unsatisfactory cervical cytology diagnosis. Obstet Gynecol 2002; 100(5 pt 1):889Y92.
34. Simavli S, Kaygusuz I, Kinay T, Cukur S. The role of gel application in decreasing pain during speculum examination and its effects on Papanicolaou smear results. Arch Gynecol Obstet 2014; 289:809Y15.
35. Gungorduk K, Ozdemir A, Gokcu M, Sanci M. Does lubrication of the vaginal speculum reduce pain during a gynaecological oncologic examination? Eur J Obstet Gynecol Reprod Biol 2015; 184:84-8.
36. Griffith WF, Stuart GS, Gluck KL. Vaginal speculum lubrication and its effects on cervical cytology and microbiology. Contraception 2005; 72: 60-4.
37. Bakker R, Peng K, Chelmow D. Speculum Lubrication and Patient Comfort: A Meta-Analysis of Randomized Controlled Trials. J Low Genit Tract Dis 2017; 21(1):67-72.
38. Charoenkwan K, Ninunanahaeminda K, Khunamornpong S, Srisomboon. Effects of gel lubricant on cervical cytology. Acta Cytol 2008;52(6):654–658
39. Köşüş A, Köşüş N, Duran M, Haltas H. Effect of liquid-based gel application during speculum examination on satisfactory level of smear examination. Arch Gynecol Obstet 2012; 285:1599–602.
40. Nunes RD, Cascaes M, Schneider IJC, Traebert J. Effects of using lubricant during the speculum examination for Pap smear collection. Diagn Cytopathol. 2018;46:1040–1044.
41. Nfor B. Lignite Zone as an Indicator to Lost Circulation Belt: A Case Study of Some Locations of Anambra State, Southeastern Nigeria. J Appl Sci Environ Manag. 2006; 10(3):31-35.
42. Arif H, Ayman J, Khalid M. Design And Determination Of The Sample Size In Medical Research. IOSR-JDMS.2014;13:21-31.
43. Chorley AJ, Marlow LAV, Forster AS, Haddrell JB, Waller J. Experiences of cervical screening and barriers to participation in the context of an organised programme: a systematic review and thematic synthesis. Psychooncology. 2017; 26:161–72.
44. Waller J, Bartoszek M, Marlow L, Wardle J. Barriers to cervical cancer screening attendance in England: a population-based survey. J Med Screen . 2009; 16:199–204.
45. Jia Y, Li S, Yang R, Zhou H, Xiang Q, Hu T. Knowledge about cervical cancer and barriers of screening program among women in Wufeng County, a high-incidence region of cervical cancer in China. PLoS One. 2013; 8:e67005.
